# Editorial: Vitamin C in Cancer and Infectious Diseases: Physiological, Biochemical and Therapeutic Interventions

**DOI:** 10.3389/fphys.2019.00734

**Published:** 2019-06-18

**Authors:** Jin Wang, Fan Wu, Christopher Corpe

**Affiliations:** ^1^Scientific Research Center, Shanghai Public Health Clinical Center, Fudan University, Shanghai, China; ^2^Nutritional Science Department, King's College London, London, United Kingdom

**Keywords:** vitamin C, cancer, infectious diseases, physiology, therapeutic intervention

Vitamin C, also known as ascorbic acid, is an essential micronutrient in the human body. Vitamin C is a co-factor for at least 15 enzymes involved in the biosynthesis of collagen and L-carnitine, Hypoxia-Inducible Factor (HIF) degradation, peptide amidation, tyrosine metabolism, and the conversion of dopamine to norepinephrine (Institute of Medicine (US) Panel on Dietary Antioxidants Related Compounds, 2000; Pei et al., 2019). It is also a detoxifier and powerful anti-oxidant, eliminating free radicals. Vitamin C is also a key regulator of immune function, cellular growth and differentiation (Ang et al., 2018). Most animals are able to synthesize vitamin C; however, 30–50 million ago humans lost the ability to synthesize the vitamin due to the development of mutations in the gulonolactone oxidase gene (Drouin et al., 2011). High concentrations of vitamin C act as a pro-oxidant, eliciting hydrogen peroxide–dependent cytotoxicity in cancer cells without adversely affecting normal cells (Chen et al., 2008). High-dose vitamin C has also been investigated in people and shown to improve the health-related quality of life of terminal cancer and infectious diseases patients (Mikirova and Hunninghake, 2014). In this research topic, we have gathered together 7 articles; 2 original research contributions and 5 review articles which are focused on the physiological role of vitamin C in health, the biochemical and molecular mechanisms of vitamin C in cancer, and the importance of vitamin C in infectious diseases.

Whether vitamin C has anti-cancer properties or not has been debated for decades (Cameron and Pauling, 1978; Moertel et al., 1985; Nauman et al., 2018). The review by Vissers and Das highlights the fact that vitamin C has been used for many years by cancer patients in an unregulated environment, either as a dietary supplement or in pharmacological doses administered by infusion. Daily vitamin C supplementation can likely reduce the incidence of gastric, esophageal, oral, pharyngeal and cervical cancer, and vitamin C-rich fruits may help prevent colon cancer and lung cancer (Pal et al., 2012). In addition, numerous reports have shown when high-doses of vitamin C are combined with conventional chemotherapy drugs the vitamin enhances the inhibition of cancer cell growth. Interestingly, the review by Vissers and Das shows pharmacologic doses of vitamin C result in oxidative stress, which preferentially targets cancer cells. Other mechanisms of action include vitamin C induced release of mitochondrial cytochrome C, leading to H_2_O_2_-mediated activation of the caspase cascade and apoptosis, and a significant decrease in tumor growth rates (Pei et al., 2016). Significant quantities of hydrogen peroxide are also generated by the autoxidation of pharmacologic levels of vitamin C and stimulation of the 2-oxoglutarate dependent dioxygenase family of enzymes (2-OGDDs) that require vitamin C as a cofactor. Support for these proposed mechanisms has come from many *in vitro* studies and xenograft animal models. With a number of early phase clinical trials currently underway, evidence for potential mechanisms of action is required to inform the most appropriate study design and choice of cancer model. Park et al. also reviewed the molecular mechanisms underlying the regulation of oxidation/reduction systems, focusing on the altered metabolomic profile in cancer cells. They suggested vitamin C alters *de novo* synthesis of GSH, resulting in alterations in the ratio of glutathione (GSH/GSSG). Alterations in GSH metabolism is further associated with increased glutathionylation of GAPDH, altered glucose metabolism, and high ROS levels and GSH oxidation. We believe the scope of this research topic provides the reader with insights into the multiple molecular mechanisms responsible for the anti-cancer properties of vitamin C.

In the present research topic, Carr and Cook investigated the use of high dose IVC in cancer patients. High-dose intravenous vitamin C (IVC) has been used for decades as a complementary, alternative, and adjuvant medicine in clinic, and has been shown to significantly prolong the survival of patients (Hoffer et al., 2015). Although IVC has been clinically accepted and used for many years, there are still many questions regarding the clinical use of IVC in cancer therapy, such as the frequency and duration of IVC treatment, the optimal route of medication, safety, interaction with chemotherapy, quality of life, and the potential mechanisms of action. Parent et al. examined the relationship between dietary intake of vitamin C, in foods, supplements and multivitamins, and the incidence of prostate cancer in a large population-based case-control study, specifically addressing the issues of cancer aggressiveness and screening patterns. Their study included 1,916 histologically confirmed prostate cancer cases and 1,985 population controls, information on a wide range of socio-demographic, lifestyle, and medical factors, including PSA screening. Consumption of 63 food items 2 years prior to diagnosis/interview was also collected, along with the use of dietary supplements. Using the Prostate Cancer and Environment Study (PROtEuS) in Montreal, Canada, their work demonstrated that vitamin C did not seem to hold promise with regard to prostate cancer prevention.

A combination of nutrition, nutraceuticals, and drugs is the preferred therapeutic approach to fight against cancer. Colorectal cancer (CRC) is classified a fatal type of cancer, and conventional first and second line chemotherapeutic regimens for CRC are based on a combination of drugs including fluoropyrimidine, oxaliplatin (Oxa), and irinotecan (Iri). Pires et al. analyzed the combination of high concentrations of vitamin C with reduced concentrations of drugs conventionally used in CRC patients and found that pharmacological vitamin C increased the efficacy of Iri and Oxa against CRC.

Another important part of this research topic is to discuss vitamin C in the treatment of infectious diseases. It is well-known that Gram-negative bacteria *Helicobacter pylori* are closely related to stomach diseases and gastric cancer. A large number of clinical reports have shown that ascorbate deficiency is associated with gastritis. In patients with *H. pylori*-infected gastritis, *H. pylori*, first discovered by Robin Warren and Barry Marshall, is one of the most common bacterial infections in gastritis, with *H. pylori* infection exceeding 50%. Gastritis and peptic ulcers are also caused by infectious pathogens that eventually develop into chronic gastritis, mucosa-associated lymphoma, and even gastric adenocarcinoma. In this research topic, Mei and Tu summarizes the relationship between vitamin C and *H. pylori* infection, and outlines potential strategies to prevent *H.pylori* infection that are based on emerging advances in our understanding of ascorbic acid physiology and pharmacology.

The final chapter in this research topic brings together a number of related dermatological studies which have shown that vitamin C can effectively promote the formation of the skin barrier via collagen synthesis, establishing a natural antioxidant barrier in the dermis, and regulating skin cell growth, differentiation, and signal transduction. Severe vitamin C deficiency leads to atopic dermatitis (AD) and porphyria (PCT). Vitamin C can be an effective drug for treating skin diseases, but the appropriate doses require further study. In this research topic, Wang et al. summarize the effects of vitamin C on five skin diseases (porphyria cutanea tarda, atopic dermatitis, malignant melanoma, and herpes zoster and postherpetic neuralgia) and emphasize the clinical application of vitamin C as an adjuvant for drugs or physical therapy for other skin diseases.

Together, this research topic gives insight into the multiple and complex roles of vitamin C, and in particular its involvement in cancer and infectious diseases. It provides guidance for therapeutic approaches and future research on the clinical use of high-dose intravenous vitamin C. Through research we need to better understand the mechanisms of vitamin C action, the optimal administration route, the appropriate dose and administration frequency. From such work we will gain insights into the clinical value of vitamin C, its role at a physiological and biochemical level, and expand the value of vitamin C in the treatment of cancer and infectious diseases. We look forward to new applied and useful data in this growing area.

## Author Contributions

JW, FW, and CC planned and edited this special topic. JW and FW wrote this editorial. JW and CC revised this editorial.

### Conflict of Interest Statement

The authors declare that the research was conducted in the absence of any commercial or financial relationships that could be construed as a potential conflict of interest.
